# Prenatal PBDE and PCB Exposures and Reading, Cognition, and Externalizing Behavior in Children

**DOI:** 10.1289/EHP478

**Published:** 2016-07-06

**Authors:** Hongmei Zhang, Kimberly Yolton, Glenys M. Webster, Andreas Sjödin, Antonia M. Calafat, Kim N. Dietrich, Yingying Xu, Changchun Xie, Joseph M. Braun, Bruce P. Lanphear, Aimin Chen

**Affiliations:** 1 Department of Environmental Health, School of Public Health, Shanxi Medical University, Taiyuan, Shanxi Province, China; 2 Division of General and Community Pediatrics, Department of Pediatrics, Cincinnati Children’s Hospital Medical Center, Cincinnati, Ohio, USA; 3 Child and Family Research Institute, BC Children’s and Women’s Hospital and Faculty of Health Sciences, Simon Fraser University, Vancouver, British Columbia, Canada; 4 Division of Laboratory Sciences, National Center for Environmental Health, Centers for Disease Control and Prevention, Atlanta, Georgia, USA; 5 Department of Environmental Health, University of Cincinnati College of Medicine, Cincinnati, Ohio, USA; 6 Department of Epidemiology, Brown University School of Public Health, Providence, Rhode Island, USA.

## Abstract

**Background:**

Prenatal polybrominated diphenyl ethers (PBDEs) and polychlorinated biphenyls (PCBs) exposures may influence children’s neurodevelopment.

**Objective:**

We examined the association of prenatal PBDE and PCB exposures with children’s reading skills at ages 5 and 8 years, Full-Scale Intelligence Quotient (FSIQ), and externalizing behavior problems at age 8 years.

**Methods:**

From 239 mother–child pairs recruited (2003–2006) in Cincinnati, Ohio, we measured maternal serum PBDE and PCB concentrations, assessed child’s reading skills using the Woodcock–Johnson Tests of Achievement III (WJ-III) at age 5 years and the Wide Range Achievement Test-4 (WRAT-4) at age 8 years, tested FSIQ using the Wechsler Intelligence Scale for Children-IV (WISC-IV), and externalizing behavior problems using the Behavioral Assessment System for Children-2 (BASC-2) at age 8 years. We used multiple linear regression to examine the association of prenatal PBDE and PCB concentrations and reading, FSIQ, and externalizing behavior problems after adjusting for covariates.

**Results:**

An increase of Sum_4_PBDEs (BDE-47, BDE-99, BDE-100, and BDE-153) by 10 times was not significantly associated with reading scores at age 5 years at the *p* = 0.05 level but was inversely associated with Reading Composite scores (β: –6.2, 95% CI: –11.7, –0.6) and FSIQ (β: –5.3, 95% CI: –10.6, –0.02) at age 8 years; it was positively associated with the score for externalizing behavior problems (β: 3.5, 95% CI: –0.1, 7.2) at age 8 years. Prenatal Sum_4_PCBs (PCB-118, -153, -138-158, and -180) was not significantly associated with a child’s reading skills, FSIQ, and externalizing behavior problems.

**Conclusion:**

Prenatal PBDE concentration was inversely associated with reading skills and FSIQ and positively associated with externalizing behavior problems at age 8 years. No significant associations were found in prenatal PCB concentration.

**Citation:**

Zhang H, Yolton K, Webster GM, Sjödin A, Calafat AM, Dietrich KN, Xu Y, Xie C, Braun JM, Lanphear BP, Chen A. 2017. Prenatal PBDE and PCB exposures and reading, cognition, and externalizing behavior in children. Environ Health Perspect 125:746–752; http://dx.doi.org/10.1289/EHP478

## Introduction

PBDEs were widely used as flame retardants in the manufacture of electronics, furniture, carpets, and textiles and are detectable in indoor dust, fish, birds, human serum, and adipose tissue. The phase-out of polybrominated diphenyl ethers (PBDEs) used in U.S. consumer products began in 2004 (Penta- and Octa-PBDE) and 2013 (Deca-PBDE), PBDEs still exist in the environment and biological samples. Polychlorinated biphenyls (PCBs), a group of structurally related organic compounds with inertness and thermal stability, were extensively used in various industrial products before 1979. Although PCBs’ manufacture was banned in 1979 due to possible adverse effects in humans, PCBs still persist in the environment and accumulate through the food chain. Currently, seafood consumption is the main exposure route in humans (Thompson and Boekelheide 2013; Xue et al. 2014).

Several previous studies have focused on the association of prenatal PBDE and PCB exposures and children’s overall cognition (Chen ZJ et al. 2014; Stewart et al. 2008; Zhao et al. 2013). In their study, Chen A et al. (2014) reported that prenatal exposure to PBDEs was associated with cognitive deficits and hyperactivity behaviors in children at age 5 years. However, few studies have assessed the influence of those chemicals on specific cognitive domains, in particular, language development and reading skills (Dzwilewski and Schantz 2015). The prenatal period is the most sensitive time window to the influence of environmental factors on cognitive development, when brain development involves neuronal proliferation, differentiation, and migration (Kang et al. 2011; Stiles and Jernigan 2010). Reading ability is central to educational attainment and academic achievement, and is predominantly influenced by the development in the brain reading region (Houston et al. 2014). Studies have reported that a child’s reading ability was impaired by prenatal maternal smoking exposure (Cho et al. 2013). Externalizing behavior problems are symptoms or signs of neurodevelopment, including hyperactivity, oppositional, and aggressive behavior, as well as conduct problems. The purpose of this study was to test the hypothesis that prenatal exposures to PBDEs and PCBs are associated with poorer reading abilities, lower intelligence, and more externalizing behavior problems in children.

## Methods

### Study Subjects

The Health Outcomes and Measures of the Environment (HOME) Study is a prospective birth cohort recruited between March 2003 and February 2006 in Cincinnati, Ohio, as previously described (Geraghty et al. 2008). The study enrolled 468 pregnant women at 16 ± 3 weeks gestation, who were ≥ 18 years old; living in Cincinnati, Ohio; not diagnosed with diabetes, hypertension, or reported infection with human immunodeficiency virus; and who did not take medicine for seizures or thyroid disorders during pregnancy. Of the 468 women, 389 remained in the study and delivered live-born singletons. For the current study, we excluded infants with congenital malformations or genetic abnormalities (*n* = 2), mothers with missing chemical measures in serum (*n* = 15), and children lost to follow-up and those who did not complete assessment at 5 or 8 years old (*n* = 131). This led to a final sample of 239 mother–child pairs for the current analysis. The HOME Study was approved by the Institutional Review Boards at the Cincinnati Children’s Hospital Medical Center (CCHMC) and the Centers for Disease Control and Prevention (CDC). All participants provided written informed consent.

### Maternal Serum Chemicals Measurement

Maternal blood samples were obtained at 16 ± 3 weeks of gestation, and sera were immediately isolated and stored at –80°C until shipment on dry ice to the National Center for Environmental Health (NCEH) at CDC for analysis. Maternal sera were analyzed for 10 PBDE congeners (BDE-17, -28, -47, -66, -85, -99, -100, -153, -154, and -183) and 36 PCB congeners (PCB-18, -28, -44, -49, -52, -66, -74, -87, -99, -101, -105, -110, -118, -128, -138-158, -146, -149, -151, -153, -156, -157, -167, -170, -172, -177, -178, -180, -183, -187, -189, -199, -196-203, -194, -195, -206, and -209) using gas chromatography/isotope dilution high-resolution mass spectrometry (Sjödin et al. 2004). All analytic runs included blank and positive quality control samples. PBDEs and PCBs were expressed as nanogram per gram lipid (ng/g lipid) because they are lipophilic compounds. Serum lipid concentrations were calculated using the Phillips formula summing total cholesterol and triglycerides (Phillips et al. 1989). Values below the limit of detection (LOD) were replaced with the LOD divided by the square root of 2 (Hornung and Reed 1990).

### Reading Ability Assessments

We assessed children’s reading skills at age 5 years using the Woodcock-Johnson Tests of Achievement-III (WJ-III) (Mather et al. 2001). For these analyses, we focused on basic reading and brief reading scores. Basic reading is a composite of Letter-Word Identification and Word Attack and measures of sight vocabulary, phonics, and structural analysis. Brief reading is a composite of Letter-Word Identification and Passage Comprehension that measures reading concepts and readiness. Letter-Word Identification measures the ability to identify letters and to read individual words. Word Attack measures skills in applying phonic and structural analysis to the reading of unfamiliar words. Passage Comprehension tests the ability to match a symbol with an actual picture of the object, to point to the picture represented by a phrase, and to identify a missing key word within a passage based on contextual cues.

At age 8 years, we measured each child’s basic academic skills to read words and comprehend sentences using the Wide Range Achievement Test 4 (WRAT-4) (Sayegh et al. 2014). The Reading Composite score was assessed in the analysis, which was calculated from the Word Reading and Sentence Comprehension subtests. Word Reading measures letter and word reading, and Sentence Comprehension measures the skills to obtain meaning from words and comprehend ideas and information in sentences. Both WJ-III and WRAT-4 items have a population mean of 100 and a standard deviation of 15.

### Neurodevelopmental and Behavioral Assessments

At age 8 years, we administered the Wechsler Intelligence Scale for Children-IV (WISC-IV) to obtain Full-Scale Intelligence Quotient (FSIQ) (Wechsler 2003), and the Behavioral Assessment System for Children-2 (BASC-2) to obtain Externalizing Problems score (Reynolds and Kamphaus 2004). The FSIQ measures cognitive function and has a population mean of 100 and standard deviation of 15. Externalizing Problems offer an assessment of a child’s adaptive and problem behaviors, which includes subscales of aggression and hyperactivity. The score for externalizing behavior problems had a population mean of 50 and a standard deviation of 10, for which higher score suggests nonoptimal behavior. All clinical assessments were performed by HOME study staff members who were trained and certified by a developmental psychologist (KY). The assessors conducted the neurobehavioral assessments without knowledge of maternal serum PBDE and PCB concentrations.

### Statistical Analysis

We used multiple linear regression to analyze the associations between maternal serum PBDE or PCB concentrations and the outcome (reading ability, FSIQ, or externalizing behavior problems) scores. We separately used the following exposure variables in the analyses: *a*) the sum of BDE-47, -99, -100, and -153 (Sum_4_PBDEs)—the four congeners detected in 91% of the HOME study samples; *b*) the sum of PCB-118, -153, -138-158, and -180 (Sum_4_PCBs)—these congeners were detected in 94% of the HOME study samples, and Sum_4_PCBs was frequently used in other U.S. cohort studies (Alm et al. 2008; Boucher et al. 2009). Considering their right-skewed distributions and more than a 100 increase in exposure ranges, log_10_-transformed Sum_4_PBDE and Sum_4_PCB concentrations were used in this analysis. Covariates for adjustment were identified based on their significant relationship with chemical concentrations and outcome scores using analysis of variance (ANOVA) or *t*-test for continuous variables, and chi-square test for categorical variables. In the final models, we adjusted for maternal age, race, education, household income, parity, marital status, smoking status, maternal fish consumption (the number of meals in which fish was consumed from baseline visit to child’s birth, maternal depression, maternal intelligence quotient (IQ), sex of the child, and the HOME score (Totsika and Sylva 2004). Maternal IQ was assessed using the Wechsler Abbreviated Scale of Intelligence; maternal depression was measured using the Beck Depression Inventory-II (Beck et al. 1996) during pregnancy. When the child was 1 year old, study staff visited them and completed the HOME Inventory describing the nurturing environment in the home (Caldwell and Bradley 1984).

We used the trend test to test the trend of each outcome by using the median value of each quartile as the independent variable. We also used the least absolute shrinkage and selection operator (LASSO) test to simultaneously perform model selection and parameter estimation of each PBDE and PCB congener’s relative contribution to the outcome scores (Tibshirani 1996). To ensure the consistency of results, the analysis was repeatedly run with all available data and with data available at both 5 and 8 years old. The statistical analysis was completed with SAS (version 9.4; SAS Institute Inc., Cary, NC), the LASSO test was performed in R (version 2.11; R Development Core Team)using glmnet. We report the regression coefficients and 95% confidence intervals (CIs) from the multiple regression analyses. The statistical significance was set at a two-sided *p*-value of 0.05.

## Results

This analysis was based on 239 mothers who were enrolled in the HOME study and provided biological samples for evaluating exposures, and their children who had outcomes of reading ability, FSIQ, and externalizing behavior problems at 5 or 8 years old. There were no significant difference in either maternal PBDE or PCB concentrations measured during pregnancy or the demographic characteristics between the 239 participants included in this analysis and those not included (data not shown).

**Figure 1:**
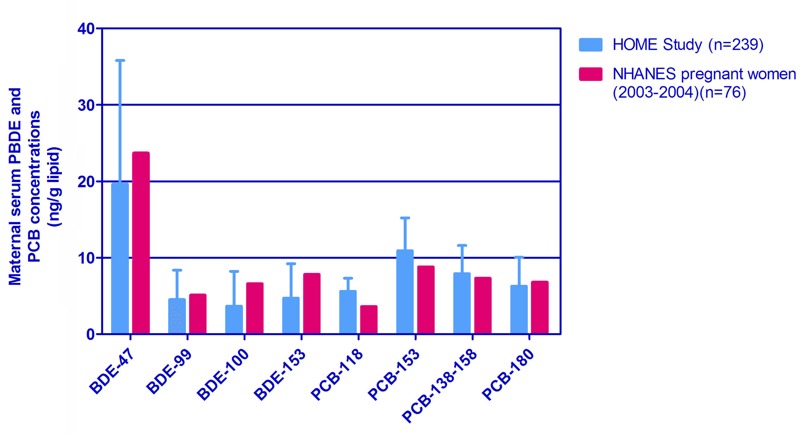
Concentrations of PBDE and PCB congeners in the pregnant women in the HOME Study and NHANES (2003–2004) (median and interquartile range). Bars represent the interquartile range (IQR) of chemical concentrations in the HOME Study. Medians of chemical concentrations in the NHANES (National Health and Nutrition Examination Survey) pregnant women (2003–2004) were referenced, which did not provide the IQR or the 25th and 75th percentiles (Woodruff et al. 2011). BDE-47, -99, -100, and -153 are the congeners of polybrominated diphenyl ethers (PBDEs); PCB-118, -153, -138-158, and -180 are the congeners of polychlorinated biphenyls (PCBs). Limit of detection (LOD) for NHANES pregnant women (2003–2004) is 4.2, 5.0, 1.4, and 2.2 ng/g lipid for BDE-47, -99, -100, and -153; 0.6 and 1.1 ng/g lipid for PCB-118, and -153, respectively; 0.4 ng/g lipid for PCB-180 and PCD-138-158.

In the HOME study, maternal median Sum_4_PBDEs concentrations (35.65 ng/g lipid) were slightly lower, the median Sum_4_PCBs (31.30 ng/g lipid) was a little higher compared with the median concentrations of the U.S. general population of pregnant women (NHANES, 2003–2004) (Sum_4_PBDEs: 43.2 ng/g lipid; Sum_4_PCBs: 26.8 ng/g lipid) (Woodruff et al. 2011) (Figure 1). Mothers were 25–34 years old (61%), non-Hispanic white (62%) with a college education or above (74%), and lived in middle- or high-income households (> $40,000/year, 76%); most of the women did not drink alcohol (56%) and did not smoke tobacco (85%) during pregnancy. Babies were born at a mean gestational age of 39 ± 5.4 weeks. Mothers who were younger than 25 years old at enrollment, were nonwhite, with a high school education or less, a household income < $40,000 per year, were not married or were living alone, were smokers or had higher depression symptom scores (> 19), and they had higher prenatal PBDE and lower PCB concentrations. Additionally, prenatal concentrations of Sum_4_PBDE and Sum_4_PCB were comparable for boys and girls (Table 1).

**Table 1:** Participant’s demographic characteristics, maternal serum PBDE and PCB concentrations, and child’s reading scores, FSIQ, and externalizing behavior problems.

Characteristics	*n* (%)	Log_10_Sum_4_PBDEs	Log_10_Sum_4_PCBs
Maternal age (years)	28.38 ± 5.75
< 25 years	57 (23.55)	1.64 ± 0.31	1.28 ± 0.24
25–34 years	150 (61.98)	1.59 ± 0.46	1.53 ± 0.24
≥ 35 years	35 (14.46)	1.40 ± 0.34	1.75 ± 0.21
Maternal education
High school or less	61 (25.21)	1.72 ± 0.38	1.38 ± 0.32
Some college or 2-year degree	62 (25.62)	1.58 ± 0.33	1.47 ± 0.20
Bachelor’s	74 (30.58)	1.50 ± 0.44	1.56 ± 0.25
Graduate or professional	45 (18.60)	1.51 ± 0.52	1.63 ± 0.27
Maternal race
Non-Hispanic white	150 (61.98)	1.52 ± 0.42	1.55 ± 0.24
Non-Hispanic black and others	92 (38.02)	1.68 ± 0.41	1.43 ± 0.32
Maternal parity
Nulliparous	111 (45.87)	1.53 ± 0.43	1.54 ± 0.31
Parity = 1	71 (29.34)	1.56 ± 0.38	1.49 ± 0.24
Parity > 1	60 (24.79)	1.66 ± 0.45	1.49 ± 0.23
Marital status
Married or living with partner	184 (76.03)	1.53 ± 0.43	1.55 ± 0.25
Not married, living alone	58 (23.97)	1.72 ± 0.36	1.38 ± 0.31
Household income
< $40,000/year	58 (23.97)	1.73 ± 0.35	1.35 ± 0.29
$40,000–79,999/year	121 (50.00)	1.57 ± 0.44	1.55 ± 0.25
≥ $80,000/year	63 (26.03)	1.42 ± 0.39	1.58 ± 0.26
Maternal alcohol drinking
Never drinking	136 (56.20)	1.56 ± 0.43	1.48 ± 0.26
< 1/month drinking	75 (30.99)	1.59 ± 0.41	1.54 ± 0.24
> 1/month or binge	31 (12.81)	1.59 ± 0.43	1.60 ± 0.37
Maternal smoking
Nonsmoking (serum cotinine < 1 ng/mL)	206 (85.12)	1.55 ± 0.42	1.52 ± 0.27
Secondhand tobacco smoking (1 ng/mL ≤ serum cotinine < 10 ng/mL)	19 (7.85)	1.63 ± 0.32	1.32 ± 0.21
Active smoking (serum cotinine ≥ 10 ng/mL)	17 (7.02)	1.91 ± 0.43	1.58 ± 0.28
HOME inventory score at 1 year home visit	39.14 ± 5.44
≥ 40	146 (64.60)	1.50 ± 0.43	1.57 ± 0.26
35–39	41 (18.14)	1.74 ± 0.39	1.46 ± 0.22
< 35	39 (17.26)	1.69 ± 0.40	1.33 ± 0.28
Maternal FSIQ	105.98 ± 15.28
Maternal depressive symptoms at 1 year home visit	9.92 ± 6.67
Minimal or mild	221 (91.70)	1.55 ± 0.42	1.53 ± 0.27
Moderate or severe	20 (8.30)	1.81 ± 0.40	1.37 ± 0.23
Child sex
Male	109 (45.04)	1.53 ± 0.42	1.52 ± 0.26
Female	133 (54.96)	1.61 ± 0.42	1.50 ± 0.28
Reading scores^*a*^
WJ-III score at age 5 years (*n *= 203)
Basic reading (a + c)	109.94 ± 17.64
Brief reading (a + b)	104.44 ± 16.83
a. Letter word	106.12 ± 15.57
b. Passage comprehension	100.66 ± 11.07
c. Word attack	118.97 ± 13.28
WRAT-4 score at age 8 years (*n *= 232)
Reading composite (d + e)	108.27 ± 15.40
d. Word reading	107.63 ± 14.24
e. Sentence comprehension	107.63 ± 17.88
FSIQ at age 8 years (*n *= 231)	102.10 ± 15.71
Externalizing problems at age 8 years (*n *= 239)	49.54 ± 9.40
Note: Data are shown as mean ± SD or *n* (%). FSIQ, Full-Scale Intelligence Quotient. ^***a***^Average score of each item without the corresponding concentrations of PBDEs and PCBs.			

Children’s scores of reading, FSIQ and Externalizing Problems were normally distributed. Reading subset items were highly correlated with each other (*r* = 0.58 for composite measures, *p* < 0.001). Reading Composite score was positively correlated with FSIQ (*r* = 0.75, *p* < 0.0001), and not correlated with Externalizing Problems score (*r* = –0.05, *p* = 0.43) at age 8 years. Reading Composite score and FSIQ were positively associated with maternal age, education, household-income, and inversely associated with maternal depression and HOME scores (see Figure S1). The score on externalizing behavior problems was negatively associated with maternal education and household income, and positively associated with maternal depression and HOME scores.

After adjusting for covariates, each increase in Sum_4_PBDEs concentration by a factor of 10 was significantly associated with a 6.6-point decrement in Sentence Comprehension score (*p* = 0.038), a 6.2-point decrement in the Reading Composite score (*p* = 0.029), and a 5.3-point decrease in FSIQ score (*p* = 0.049). It was marginally associated with a 3.5-point increase in Externalizing Problems score (*p* = 0.057) at 8 years of age (Table 2). Sum_4_PCBs concentration as a continuous variable was positively associated with children’s reading scores, FSIQ and Externalizing Problems at age 8 years, but none of them was statistically significant (Table 2). The Sum_4_PCBs concentration was correlated with maternal fish consumption during pregnancy (*r* = 0.38, *p* < 0.0001), whereas the Sum_4_PBDEs concentration was not (*r* = 0.05, *p* = 0.38). Nonsignificant interaction was found between the fish consumption and serum Sum_4_PCBs on child’s reading abilities, FSIQ, and externalizing behavior problems in the HOME study (data not shown).

**Table 2:** Adjusted β coefficients (95% CIs) in the multiple linear regressions of child’s reading scores, FSIQ, and Externalizing Problems score with prenatal serum Sum_4_PBDEs and Sum_4_PCBs concentrations (ng/g lipid).

Chemicals	WJ-III score (*n *= 203) at age 5 years		WRAT-4 score (*n *= 232) at age 8 years	WISC-IV (*n *= 231) at age 8 years	BASC-2 (*n *= 239) at age 8 years
	Basic reading	Brief reading	Reading composite	FSIQ	Externalizing problems
Log_10_Sum_4_PBDEs	–3.1 (–10.1, 3.9)	–2.8 (–9.9, 4.3)	–6.2 (–11.7, –0.6)*	–5.3 (–10.6, –0.1)*	3.5 (–0.1, 7.2)^#^
Log_10_Sum_4_PCBs	6.7 (–4.8, 18.3)	6.5 (–5.2, 18.2)	7.0 (–2.2, 16.2)	1.1 (–7.9, 10.0)	0.5 (–5.7, 6.7)
Note: Adjusted for maternal age, education, race, IQ, household-income, parity, marital status, maternal smoking (serum cotinine concentrations), depression, fish consumption, child’s sex, and HOME score. BASC-2, Behavioral Assessment System for Children-2; CIs, confidence intervals; FSIQ, Full-Scale Intelligence Quotient; WISC-IV, Wechsler Intelligence Scale for Children-IV; WJ-III, Woodcock-Johnson Tests of Achievement-III; WRAT-4, Wide Range Achievement Test-4; *p < 0.05, #*p* < 0.10.					

**Figure 2:**
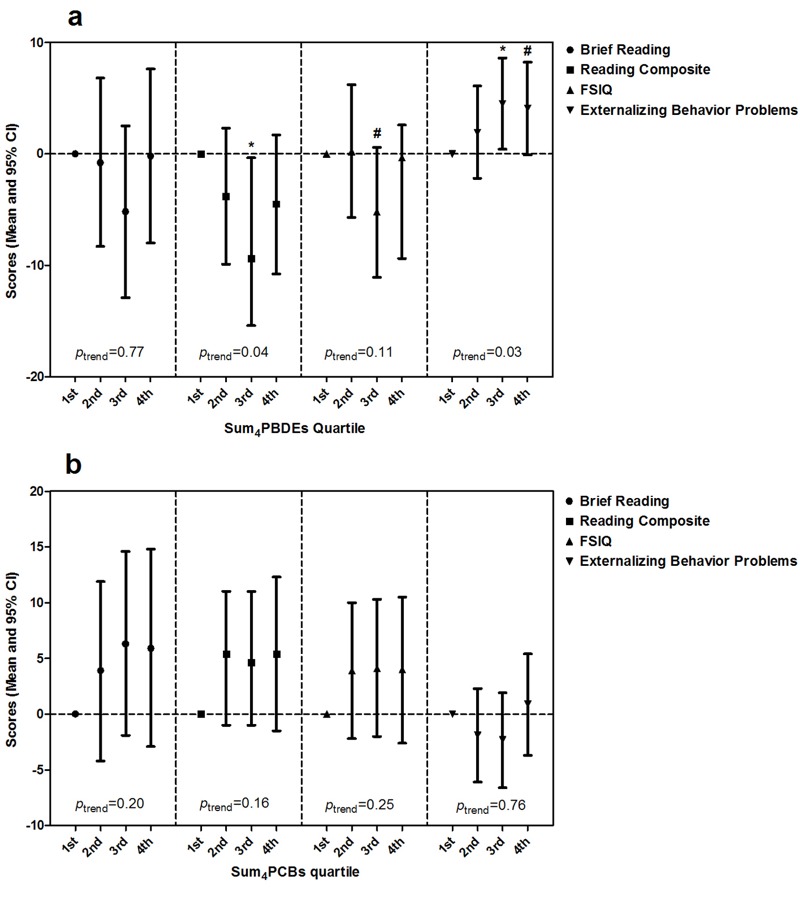
Trend and association of child’s reading scores, FSIQ, and Externalizing Problems scores with prenatal Sum_4_PBDEs and Sum_4_PCBs concentrations quartiles. (*a*) The trend and association of child’s reading scores, FSIQ, and externalizing behavior problems scores with prenatal Sum_4_PBDEs; (*b*) The trend and association of child’s reading scores, FSIQ, and externalizing behavior problems scores with prenatal Sum_4_PCBs. The quartile cutoffs were < 20.70, 20.70–35.64, 35.65–76.00, and ≥ 76.00 ng/g lipid for Sum_4_PBDEs, and < 21.50, 21.50–31.29, 31.30–42.80, and ≥ 42.80 ng/g for Sum_4_PCBs, respectively. The score in the 1st quartile is the reference. Note: Adjusted for maternal age, education, race, IQ, household income, parity, married status, smoking (maternal serum cotinine), fish consumption, depression, and child sex, and HOME score. FSIQ, Full-Scale Intelligence Quotient; *p < 0.05, ^#^ *p *< 0.10.

Trend tests based on quartiles indicated that as the median Sum_4_PBDEs increased in the quartiles, Reading Composite score was significantly decreased (*p* = 0.04), Externalizing Problems score was statistically increased (*p* = 0.03). No significant trend was found in Brief Reading score and FSIQ with Sum_4_PBDEs; neither trend nor association was significant between Sum_4_PCBs quartiles and reading scores at ages 5 and 8 years, FSIQ, and externalizing behavior problems scores at age 8 years (Figure 2).

The specific analysis for each PBDE or PCB congener demonstrated that an increase of BDE-99 by a factor of 10 was marginally associated with a 5.1-point decrease (95% CI: –10.3, 0.2) in FSIQ at age 8 years (*p* = 0.058). An increase of BDE-100 by a factor of 10 was significantly associated with a 6.0-point decrease (95% CI: –11.8, –0.3) in Sentence Comprehension score (*p* = 0.039), and a 5.7-point decrease (95% CI: –10.8, –0.6) in Reading Composite score (*p* = 0.028) at age 8 years. An increase of BDE-154 by a factor of 10 was significantly associated with a 6.3-point decrease (95% CI: –11.6, –0.9) in Sentence Comprehension score (*p* = 0.022), a 5.4-point decrease (95% CI: –10.1, –0.7) in Reading Composite score (*p* = 0.026), and a 3.9-point increase (95% CI: 0.8, 6.9) in the score for externalizing behavior problems (*p* = 0.014) at age 8 years. No significant associations were found between PCB congeners and children’s reading abilities, FSIQ and Externalizing Problems (Table 3). The results were similar when we ran the analysis with all available data and data available at both 5 and 8 years of age. The findings of the LASSO analysis were consistent with the results of the multiple linear regression, and are seen in Table S1.

**Table 3:** Adjusted β coefficients (95% CIs) in the multiple linear regressions of child’s reading scores, FSIQ and Externalizing Problems score with prenatal serum concentrations of PBDE and PCB congeners (ng/g lipid).

Chemicals	WJ-III score (*n *= 203) at age 5 years		WRAT-4 score (*n *= 232) at age 8 years	WISC-IV score (*n *= 231) at age 8 years	BASC-2 score (*n *= 239) at age 8 years
	Basic reading	Brief reading	Reading composite	FSIQ	Externalizing problems
Log_10_ BDE-47	–1.2 (–8.0, 5.6)	–0.9 (–7.7, 6.0)	–4.8 (–10.1, 0.6)	3.7 (–8.8, 1.5)	2.1 (–1.4, 5.6)
Log_10_ BDE-99	–3.8 (–10.6, 3.0)	–3.2 (–10.0, 3.7)	–4.2 (–9.7, 1.3)	–5.1 (–10.3, 0.2)^#^	2.8 (–0.8, 6.4)
Log_10_ BDE-100	–3.1 (–9.7, 3.4)	–3.1 (–9.7, 3.5)	–5.7 (–10.8, –0.6)*	–4.6 (–9.5, 0.2)^#^	2.6 (–0.7, 6.0)
Log_10_ BDE-153	–4.8 (–10.5, 1.0)	–4.7 (–10.6, 1.1)	–5.4 (–10.1, –0.7)*	–3.6 (–8.2, 0.9)	3.9 (0.8, 6.9)*
Log_10_ PCB-118	2.0 (–7.8, 11.7)	1.2 (–8.7, 11.1)	3.8 (–4.2, 11.8)	–0.5 (–8.2, 7.2)	–1.2 (–6.6, 4.1)
Log_10_ PCB-153	6.3 (–4.8, 17.4)	7.1 (–4.1, 18.4)	6.3 (–2.5, 15.1)	–0.1 (–8.7, 8.5)	0.8 (–5.1, 6.7)
Log_10_PCB-138-158	6.2 (–4.5, 16.8)	5.5 (–5.3, 16.3)	3.5 (–5.1, 12.0)	0.6 (–7.7, 8.9)	0.9 (–4.8, 6.7)
Log_10_ PCB-180	4.4 (–6.3, 15.2)	6.0 (–4.8, 16.8)	6.8 (–1.6, 15.2)	0.7 (–7.5, 8.9)	0.8 (–4.9, 9.4)
Note: Adjusted for maternal age, education, race, IQ, household-income, parity, marital status, maternal smoking (serum cotinine concentrations), depression, fish consumption, child sex, and HOME score. BASC-2, Behavioral Assessment System for Children-2; CIs, confidence intervals; FSIQ, Full-Scale Intelligence Quotient; WISC-IV, Wechsler Intelligence Scale for Children-IV; WJ-III, Woodcock-Johnson Tests of Achievement-III; WRAT-4, Wide Range Achievement Test-4; *p < 0.05, #*p *< 0.10.					

## Discussion

In this study, we comprehensively assessed various aspects of a child’s neurodevelopment, including reading abilities, intelligence and behavior, and highlighted that the reading ability was an important aspect of neurodevelopment, which was influenced by prenatal chemical exposure. After adjustment, prenatal PBDE concentration was inversely associated with reading skills and FSIQ, and positively associated with externalizing behavior problems in children at age 8 years. Prenatal PBDE concentration was not associated with reading skills at age 5 years; Prenatal PCB concentration was not statistically associated with reading abilities, FSIQ and externalizing behavior problems in children at age 5 or 8 years. Prenatal PBDE and PCB concentrations were measured in maternal serum samples at approximately 16 weeks of gestation, when blood volume expansion and hyperlipidemia status have not been established (Bloom et al. 2007; Faupel-Badger et al. 2007), thus the impact of physiological variation during pregnancy on prenatal serum PBDE and PCB concentrations is less important in this study.

No evidence is reported on the association of prenatal PBDE and PCB concentrations with child’s reading ability so far. This study highlights reading ability as a key outcome, and found that prenatal PBDE concentration was inversely associated with child’s reading ability at age 8 years after adjustment for covariates, but was not associated with reading ability at age 5 years. Reading ability is an aspect of neurodevelopment, which was significantly influenced by the neurodevelopment maturity, such as brain area and volume (Spann et al. 2014), the ratio of neuron to glial cells, cell integrity and interneuronal communication underlying the frontal and temporal cortices (O’Muircheartaigh et al. 2014). The results in the HOME study showing the association of prenatal PBDE with decrements in FSIQ and increment in externalizing behavior problems were remarkably consistent with previous studies. In the HOME study, the decrement in FSIQ [–5.3 (95% CI: –10.6, –0.1)] and increment in externalizing behavior problems [3.5 points (95% CI: –0.1, 7.2)] at age 8 years with a 10-fold increase in maternal serum Sum_4_PBDEs concentration are consistent with our previous report for FSIQ (β: –4.5, 95% CI: –8.8, –0.1) and externalizing behavior problems (β: 2.6, 95% CI: –0.4, 5.5) at age 5 years with maternal serum BDE-47 concentration (Chen A et al. 2014), and are also consistent with other two reports [California farming community cohort: FSIQ (β: –4.7, 95% CI: –9.4, 0.1) and Attention Deficit/Hyperactivity Disorder (ADHD) index (β: 2.9, 95% CI: –0.7, 5.2) at age 7 years with maternal Sum_4_PBDEs; World Trade Center cohort: FSIQ (β: –5.5, 95% CI: –10.8, –0.2) at age 4 years related to cord BDE-47 (derived from original estimate of natural log BDE-47)] (Eskenazi et al. 2013; Herbstman et al. 2010). Maternal serum PBDE concentration was associated with more externalizing behavior problems (BDE-100, β: 0.31), and worse fine manipulative abilities (BDE-154, β: –0.30) in 62 healthy Dutch children aged 5–6 years (Roze et al. 2009).

Several mechanisms underlying PBDE-mediated developmental neurotoxicity have been suggested in rats and mice neonatal exposure to PBDE (Johansson et al. 2008; Viberg et al. 2003, 2005, 2007; Zhang et al. 2013). BDE-47 and BDE-153 changed hippocampus morphology and ultra structure in rats (He et al. 2009; Zhang et al. 2013). BDE-47 and BDE-99 damaged rats or mice’s neuron cytoskeletal formation and neuronal maturation by affecting Ca^2+^/calmodulin-dependent protein kinase II, synaptophysin and cytoskeletal protein expression, decreasing expressions of brain-derived neurotrophic factor and anti-apoptotic bcl-2 mRNA, and protein (Alm et al. 2008; Blanco et al. 2011; Viberg 2009). PBDEs induced oxidative stress, DNA damage and apoptosis (He et al. 2008; Tagliaferri et al. 2010), disrupted intercellular Ca^2+^ homeostasis (Gassmann et al. 2014), affected neuron cell signal transduction (Fan et al. 2010), and voltage-gated sodium channels *ex vivo* (Xing et al. 2010). Moreover, PBDEs suppressed thyroid receptor-mediated transcription (Ibhazehiebo et al. 2011), impaired rats’ synaptic plasticity (Xing et al. 2009), and inhibited human neural progenitor cells migration and differentiation into neurons and oligodendrocytes (Schreiber et al. 2010). All of the above provided potential mechanisms underlying PBDE-mediated developmental neurotoxicity.

PCBs are among the most well-studied endocrine-disrupting chemicals; they can cause rat pups’ hyperactivity (Lesmana et al. 2014), induce oxidative stress, and increase apoptosis in the developing rat brain (Yang and Lein 2010). In the HOME study, prenatal Sum_4_PCBs concentration (median: 31.30 ng/g lipid) was not associated with reading skills, FSIQ, and externalizing behavior problems in children at age 8 years after adjusting for maternal fish consumption and other covariates. These findings were inconsistent with the Oswego cohort study that showed an inverse association between placenta Sum_4_PCBs concentration (1.5 ng/g wet) and FSIQ (β = –0.167, *p* = 0.021) and verbal IQ (β = –0.213, *p* = 0.003) in 9-year-old children (Stewart et al. 2008). The LASSO test also revealed positive associations of Sum_4_PCBs or some PCB congeners with child’s neurodevelopment in this study. Consumption of contaminated fish and other food is the main source of human exposure to PCBs (Braun et al. 2014). Fish contains beneficial nutrients for fetal development and maternal health, including polyunsaturated fatty acids (PUFAs) and others, but also may contain harmful contaminants such as PCBs and methylmercury (MeHg). In the HOME study, maternal serum PCB concentrations were positively correlated with fish consumption, yet nonsignificant interaction was found between maternal serum Sum_4_PCBs and fish consumption on outcomes.

This study has notable strengths and limitations. We comprehensively focused on children’s reading abilities, an often overlooked facet of neurodevelopment, and FSIQ and externalizing behavior problems, in a prospective birth cohort and assessed chemical exposure during the sensitive prenatal period using maternal serum chemical concentrations. We also successfully followed up children into school age when neurodevelopment measures stabilize. We tested the trend of outcome with maternal serum PBDE and PCB concentrations in quartile analysis. One limitation is that we did not have the measurements of serum PUFAs, the omega-3 docosahexaenoic acid, and MeHg in this analysis. These may be correlated with co-exposure to PCBs through fish consumption and neurodevelopmental outcomes and thus may confound our analysis. Another limitation is that there may be a possible random finding in multiple comparisons without corrections, which cannot be entirely ruled out here. While there are statistical methods such as Bonferroni correction to control for multiple comparisons, they tend to be stringent and neglect the correlation between neurodevelopmental outcomes. Finally, we had limited sample size at ages 5 years and 8 years that limited our statistical power and precision.

In conclusion, prenatal PBDE concentration was inversely associated with children’s reading abilities and FSIQ at age 8 years and positively associated with externalizing behavior problems at age 8 years. Prenatal PCB concentration was not significantly associated with child’s reading abilities, FSIQ, and externalizing behavior problems. These findings highlight the importance of understanding the potential effect that exposure to persistent organic chemicals during pregnancy has on children’s neurodevelopment during early childhood. Our results indicate that there is a need to protect children’s neurodevelopment from environmental exposure to contaminants during the entire gestation period by minimizing exposure to persistent organic chemicals. Additionally, reading ability is an aspect of neurodevelopment and should be assessed in further studies.

## Supplemental Material

(706 KB) PDF
