# Supplementary material for: Prenatal PBDE and PCB Exposures and Reading, Cognition, and Externalizing Behavior in Children
Source: Environ Health Perspect. 2016 Jul 6;125(4):746–52. doi: 10.1289/EHP478 (PMC5381998; doi:10.1289/EHP478)
Supplement: (706 KB) PDF [file EHP478.s001.acco.pdf]

*Environ Health Perspect*
DOI: 10.1289/EHP478

**Note to readers with disabilities:** *EHP* strives to ensure that all journal content is accessible to all readers. However, some figures and Supplemental Material published in *EHP* articles may not conform to 508 standards due to the complexity of the information being presented. If you need assistance accessing journal content, please contact ehp508@niehs.nih.gov. Our staff will work with you to assess and meet your accessibility needs within 3 working days.

# Supplemental Material

# Prenatal PBDE and PCB Exposures and Reading, Cognition, and Externalizing Behavior in Children

Hongmei Zhang, Kimberly Yolton, Glenys M. Webster, Andreas Sjödin, Antonia M. Calafat, Kim N. Dietrich, Yingying Xu, Changchun Xie, Joseph M. Braun, Bruce P. Lanphear, and Aimin Chen

## Table of Contents

**Table S1.** Coefficients in LASSO test of maternal chemical concentrations to children's reading scores, FSIQ and Externalizing Problems score at age 8 years

**Figure S1.** Children's Reading Composite scores at age 8 years by maternal participants and children's demographic characteristics in the HOME Study

Table S1. Coefficients in LASSO test of maternal chemical concentrations to children's reading scores, FSIQ and Externalizing Problems score at age 8 years

| Models | Chemicals | Reading Composite | FSIQ | Externalizing Problems |
|---|---|---|---|---|
| Model 1 | $Log_{10}Sum_4PBDEs$ | -5.6 | -3.8 | 2.1 |
| | $Log_{10}Sum_4PCBs$ | 6.5 | 0.5 | - |
| Model 2 | $Log_{10}$BDE-47 | - | - | - |
| | $Log_{10}$BDE-99 | - | -4.1 | 0.5 |
| | $Log_{10}$BDE-100 | -3.8 | - | - |
| | $Log_{10}$BDE-153 | -1.6 | -0.5 | 2.2 |
| | $Log_{10}$PCB-118 | 1.0 | - | -0.3 |
| | $Log_{10}$PCB-138-158 | - | - | - |
| | $Log_{10}$PCB-153 | 6.2 | - | - |
| | $Log_{10}$PCB-180 | 0.5 | 1.3 | - |

^: Adjusted for maternal age, education, race, IQ, household-income, parity, marital status, maternal smoking (serum cotinine concentrations), depression, fish consumption, child sex, and HOME score. It can not yield 95% Cis when selecting variables and estimating parameters.

Model 1: LASSO regression with $Log_{10}Sum_4PBDEs$ and $Log_{10}Sum_4PCBs$ after adjustment.

Model 2: LASSO regression with $Log_{10}$ transformed PBDE congeners (BDE-47, -99, -100, and -153) and PCB congeners (PCB-118, -138-158, -153, and -180) after adjustment.

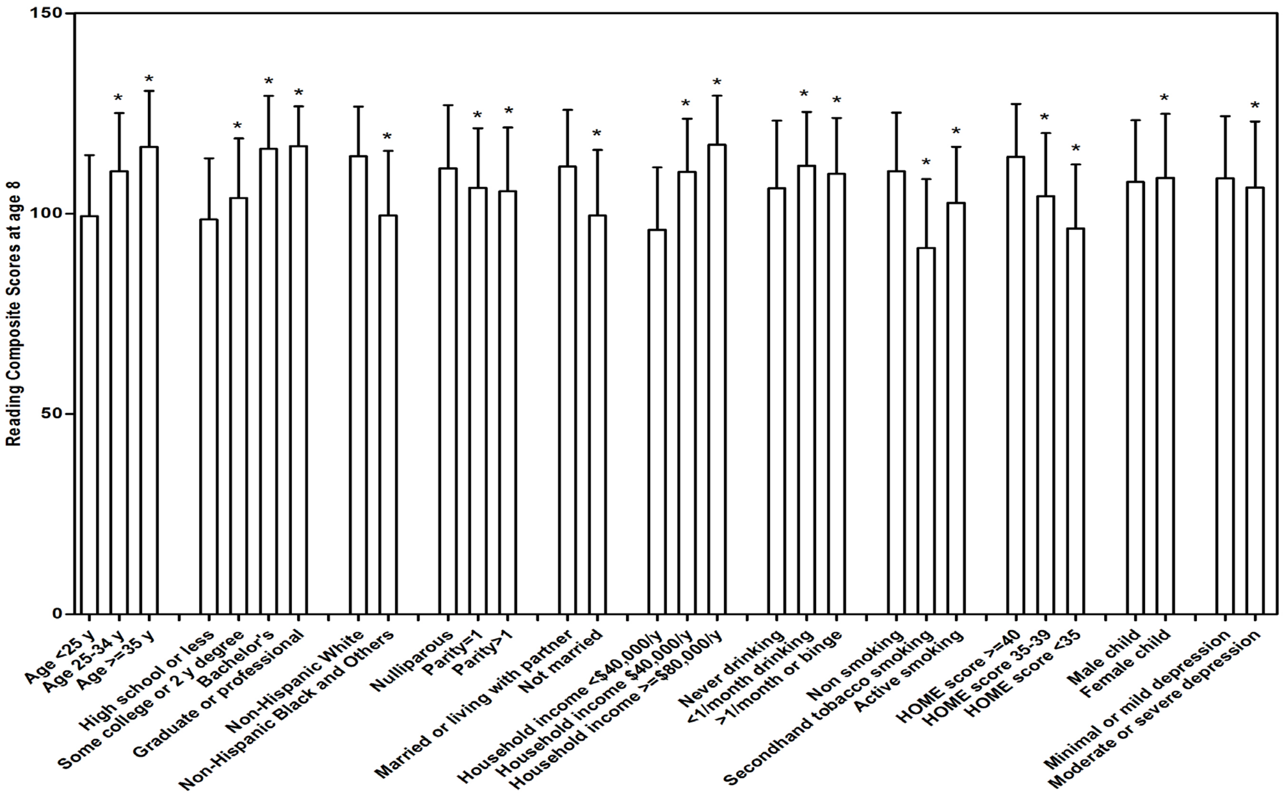


Figure S1. Children's Reading Composite scores at age 8 years by maternal participants and children's demographic characteristics in the HOME Study ("*": $p < 0.05$ comparing to the first subgroup given in this graph.)
